# Fused federated learning framework for secure and decentralized patient monitoring in healthcare 5.0 using IoMT

**DOI:** 10.1038/s41598-025-06574-w

**Published:** 2025-07-07

**Authors:** Bassam Almogadwy, Abdulrahman Alqarafi

**Affiliations:** College of Computer Science and Engineering, Taibah University, 42353 Medina, Saudi Arabia

**Keywords:** Healthcare 5.0, Internet of things, Health care, Federated learning, Data science, Medical data, IoT, Artificial intelligence, IoMT, Diseases, Health care, Medical research

## Abstract

Federated Learning (FL) enables artificial intelligence frameworks to train on private information without compromising privacy, which is especially useful in the medical and healthcare industries where the knowledge or data at hand is never enough. It paved the way for a substantial amount of study because of the high degree of communication efficacy it possessed, which is connected to dispersed training issues. The major goal of this paper is to shed light on how FL approaches might be adapted and put to use in several aspects of healthcare, including medicine discovery, medical assessment, digital health management, and the forecasting and identification of disease. This article presents a comprehensive and in-depth study of the data about fused federated learning in healthcare version 5.0. The purpose of this research is to develop a Healthcare 5.0 monitoring system by utilizing a fused federated learning approach integrated with RTS-DELM. It gives medical practitioners the ability to monitor patients through the use of various medical sensors and to take remedial action at regular intervals. The approach is shown to be successfully improved by the use of the recommended system, which is intended for healthcare monitoring. This paper introduces a novel framework leveraging Fused Federated Learning (FFL) integrated with IoMT devices aimed at securely monitoring patient health data in a decentralized manner. This study introduces a novel integration of Real-Time Sequential Deep Extreme Learning Machine (RTS-DELM) and Fused Federated Learning (FFL) for secure and decentralized chronic kidney disease diagnosis within Healthcare 5.0. The proposed approach efficiently aggregates data from distributed Internet of Medical Things (IoMT) devices, enhancing predictive accuracy while maintaining patient privacy. Experimental validation demonstrates significant improvements, achieving an accuracy rate of 98.21%, thereby showcasing superior performance over existing federated learning methodologies.

## Introduction

Current healthcare monitoring systems, despite technological advancements, have critical challenges like failure in real-time monitoring of diseases, inconsistent prediction accuracy, poor linking of heterogeneous data sources, and privacy vulnerabilities caused by centralized data management. These limitations impact timely diagnosis and management of chronic diseases which puts patients at risk. To make progress on these significant flaws, this study puts forward a new generation of decentralized healthcare monitoring systems combining federated learning with data fusion processes that are both private and highly accurate. Healthcare 5.0 refers to the next-generation digital healthcare paradigm characterized by intelligent decentralization, real-time data integration through Internet-of-Medical-Things (IoMT), personalized patient care, and advanced predictive analytics. It utilizes emerging technologies like federated learning, blockchain^1^, artificial intelligence, and secure cloud platforms to transform healthcare service delivery^2^.

The healthcare systems across the globe are indulged in crucial problems, the most evident of which is the lack of privacy and security of centralized patient data management, which further makes the challenge of predictive capabilities in real-time, and the challenge of integrating different medical information very hard. Typically, the existing federated learning approaches are still burdened with lots of important drawbacks, which include a substantial level of susceptibility to inference attacks a low level of computational efficiency for real-time monitoring, and eventually improper and untrained use of heterogeneous data. Similarly, the conventional machine learning approaches also suffer from not being able to keep the patients’ healthcare information confidential. Addressing these immense practical issues in the real world is the impetus for the introduction of a novel Fused Federated Learning (FFL) scheme that capitalizes on both Federated Learning (FL) and Real-Time Sequential Deep Extreme Learning Machine (RTS-DELM) and will guarantee privacy, increase accuracy, and enable real-time and decentralized health monitoring that concentrates on kidney disease prediction.

The recent technical discoveries in the fields of IoT and AI, as well as their applications in healthcare, are improving people’s overall quality of life and lengthening their lifespans^3^. For example, the existing COVID-19 epidemic is placing new stresses on the healthcare system and causing a desire for it. Technological solutions developed with the aid of AI and IoT may help give assistance and treatment to medical physicians and other healthcare professionals in meeting these demands^4–6^. These kinds of solutions that are supported by AI and the Internet of Things couldn’t have come at a more appropriate moment. To train automated diagnostic systems how to look for specific illness signs and patterns, medical data including imaging and pathology data are being employed. After that, the automated diagnostic systems will learn how to reliably and precisely diagnose and anticipate illnesses that occur within the human body^7–9^.

Data-driven medical applications have recently arisen as a potentially useful method for the construction of accurate and extensible prediction algorithms derived from medical information. As a result, they have garnered a significant amount of attention from a variety of sectors, including academia and business^10^. Inevitably, this has resulted in an improvement in the standard of healthcare provision^11^. Nonetheless, such data-driven medical apps are still struggling with low acceptance due to their inability to fulfill demanding safety, security, and Service Quality (QoS) standards. This is primarily the case because such systems have constraints. In addition, people, patients, and organizations that control the information are hesitant to share it with an external third party because they are concerned about the privacy of their information being compromised. These conversations are still going on, and there is a requirement for developing novel models for the exchange of data^12^.

Creating effective mining algorithms to assess medical data is crucial for advancing illness research, developing effective treatments, and enhancing the well-being of patients. Techniques from the field of machine learning have found use in many different areas, such as medical diagnosis, language analysis, and pattern recognition. The accuracy level of machine learning-based models depends on large-sized training sets, which in healthcare can mark the difference between life and death for a patient. Very often, large volumes of data that can be collected through a trustworthy cloud server form part of most common centralized training procedures, which in turn might violate major consumer privacy interfaces, particularly in healthcare^13^.

In recent years, a significant focus has emerged on the federated learning paradigm because of the important potential that it holds for facilitating easy learning with sensitive data that is both scattered and dispersed across numerous locations. It is an approach that enables one to create a unified architecture across the globe, using a centralized system, but at the same time, helping safeguard the data from its sources in the local organizations. This is in contrast to aggregating information from multiple sources at the same time or relying on finding through traditional methods and then replication. A decentralized machine learning platform for the Internet of Things, federated learning (FL) allows multiple nodes to jointly train a single model on various localized data without compromising any sensitive information^14^. This would benefit the intelligent healthcare system since sensitive information about patients is not released without obtaining their consent^15^. Fused Federated Learning (FFL) combines federated learning’s decentralized training approach with data fusion techniques. This integration improves model accuracy and generalization capability by aggregating insights from diverse datasets without compromising privacy.

Healthcare 5.0^16–19^ is a decentralized system that relies heavily on 5G connections between medical equipment. As more and more devices connect to the internet, more and more data will be produced that can be used by AI. In doing so, not only will the health and well-being of individual patients be prioritized, but also the quality of life and well-being of people all over the world. Error-free and minimally disrupted information transfer is a top priority in Healthcare 4.0. The healthcare 5.0 revolution includes the introduction of new technologies like automation and artificial intelligence (AI), both of which have the potential to significantly impact the economy.

Accurate and systematic illness forecasting, online patient monitoring and surveillance, smart robotics, and smart therapy, which may include virtual coaching for anxious patients, all fall under the purview of the healthcare industry’s fifth generation of intelligence. All types of intellectual growth can be included under the umbrella term “artificial intelligence” (AI)^20^. It’s a way to talk about how machine learning methods can predict results without any human intervention. The fast growth and enhancement of technical capabilities within the medical business led to the conception of a “smart health system.

To overcome the aforementioned issues, this work combines FL and advanced encryption to create a healthcare 5.0 system that is secure and protects patient privacy. The following is a list of the most important contributions that can be attributed to this study;

- How effectively can federated learning and data fusion techniques be combined to enhance disease prediction accuracy in decentralized healthcare environments?
- Can integrating advanced encryption with federated learning adequately protect patient privacy during real-time data transmission?
- What are the specific advantages of Fused Federated Learning over traditional federated learning approaches in healthcare monitoring?

The primary contribution of the present research project is the innovative combination of Federated Learning and the Real-Time Sequential Deep Extreme Learning Machine (RTS-DELM) - coining the term Fused Federated Learning (FFL). Traditional federated learning techniques that only aggregate model parameters do not consider the incorporation of real-time data fusion techniques that are innate to DELM, thus our approach acts as a catalyst in amplifying the accuracy and efficiency of healthcare monitoring systems. The real difference is in this blend: RTS-DELM’s quick computation features and capacity for adjusting to real-time data, as well as the very powerful decentralized privacy-preserving aggregation abilities of FL. This merger on the one hand, takes away the shortcomings of each of the methods when they are used in isolation, while on the other hand, it causes the emergence of a strong hybrid system, that can give secure, real-time, and accurate predictions in sensitive healthcare environments.

### Research questions

- How effectively can federated learning and data fusion techniques enhance predictive accuracy in decentralized healthcare?
- Can advanced encryption with federated learning adequately protect patient privacy during real-time data transmission?
- What specific advantages does the fusion of RTS-DELM and federated learning provide over traditional federated learning approaches?

This paper begins with the discussion above. The related work summaries are then reviewed in Section “2”. Section “3” provides the proposed methodology in detail, whereas the simulation results with the proposed approach are given in Section “4”. Lastly, conclusions gathered from the study are then reflected upon in Section “5”.

## Literature review

Ihnaini et al.^21^ proposed a strategy for predicting the likelihood of developing diabetes that makes use of machine learning techniques. The suggested method can improve the suggested system’s efficacy in properly forecasting and advising this life-threatening situation by merging information, which reduces unneeded pressure on the system’s computational resources. At last, a model for predicting the onset of diabetes is developed using an ensemble machine-learning strategy.

Data may be readily sent over numerous networks in today’s world, which enables professionals and businesses to maximize their use of available resources while simultaneously satisfying the needs of society in the field of medicine. Because of the Internet of Things, users now have access to healthcare services that are both reliable and highly effective. The accurate monitoring of the needs of the community in terms of medical treatment has been facilitated by the utilization of intelligent sensors. Wearable technology makes it possible to keep track of a wide variety of physiological processes. Such devices could be installed to monitor various bodily systems. Through such monitoring, proper care could be administered to the patients. The data captured by such devices can then be analyzed, integrated, and processed to get useful insights so that appropriate results can be made for accurate disease prediction^22^. Khan et al.^23^ recommended alternative medical facilities for elderly people that were centered on the actual requirements and issues faced by the patients. The researchers implemented several forms of machine learning to better meet the fundamental requirements of aged healthcare.

### Federated learning methods in healthcare

Xu et al.^24^ provided an outline of federated learning strategies, with an emphasis on those utilized in biomedicine. This article discusses and describes the overall solutions to the empirical challenges, equipment failures, and confidentiality issues that are intrinsic in federated learning with an emphasis on the potential and repercussions for healthcare. Siddiqui et al.^25^ utilized a deep learning framework to forecast the phases of breast cancer, and one of the techniques they used was data fusion. They utilized a decision-based fusion approach to get a higher degree of precision using the presented methodology.

### Security and privacy issues in IoMT

Since the introduction of artificial intelligence, it has been common practice to use AI applications to aid in illness diagnosis, enhance disease prognosis, and reduce patient treatment periods. Dai et al.^26^ converted the challenge of hospitalization prediction into an issue of supervised categorization, which led to a considerable set of possible savings in medical spending budgets. Tariq et al.^27^ created a method based on multi-dimensional fusion and artificial intelligence to predict the severity of COVID-19. Sedik et al.^28^ identified the coronavirus by using convolutional Neural Networks (NN) and convolutional LSTM methods. Additionally, they devised a method for the extraction of features to increase the amount of information that was available.

###  Data fusion techniques in healthcare applications

Qayyum et al.^29^ proposed a strategy based on clustered FL for interpreting medical visual input at the edge. This would allow remote medical facilities to benefit from heterogeneous information while preserving patients’ privacy. Brisimi et al.^30^ used FL to address scattered sparse Support Vector Machine problems, which allowed them to provide accurate therapy predictions for individuals suffering from cardiovascular diseases. However, these centralized training systems require the aggregation of personal medical data into a centralized database, which is a barrier to privacy concerns. On the contrary, federated learning appears as a distributed system that enables collaborative learning while leaving all personal information natively, thus giving a private solution to combining different flavors of healthcare information on edge devices. This architecture can be considered as a hybrid of distributed and centralized learning. In recent years, a large number of research works have emerged that intend to apply federated learning to the domain of smart healthcare. Typically, these works highlight how federated learning can be supportive of decentralized collaboration by healthcare institutions in building models that would not necessarily have required disclosure of patient data. This approach has gained increased exploration as a means to better personalized care, optimal treatment outcomes, and protection of data security in the health system. Consequently, federated learning has been one of the significant key transformative technologies for the future of smart healthcare.

Through their research, Ghadi et al.^31^ looked into the possibility of implementing blockchain technology in the Internet of Medical Things (IoMT) to improve the security and privacy of healthcare data. The characteristics of blockchain, such as decentralization and encryption, data integrity, confidentiality, and transparency will ensure that it will be a proper solution to the data management of IoMT. The results of this research also point to the potential role of blockchain in minimizing risks such as the unauthorized access and disclosure of medical records, as well as whether the health service provider complies with HIPAA regulations. Besides, Ghadi et al. stressed the capability of blockchain to enhance communication between systems thus allowing healthcare data management to be more secure and efficient.

Mazhar et al.^32^ delved into the transformational effect that Artificial Intelligence (AI) has had on neurosurgery and neurology, where it has shown promising results in diagnosis, treatment, and patient care improvements. AI-based developments, such as machine learning for disease forecasting and medical wearables for continuous health status monitoring, have made prevention and resource utilization much better. Besides, AI technologies have ushered in a new era of surgical robots that can be both precise and operated from a distance, thus reducing the risk of human errors in neurosurgical processes. Mazhar et al. in their study also looked into AI’s role in drug discovery as well as patient engagement done through chatbots and virtual assistants. The paper shows the application of blockchain technology to deal with data security and management issues, which ensures the safe and effective use of AI in neurological healthcare.

Fused Federated Learning (FFL) has come to the forefront as a possible solution to address problems in the healthcare field, especially that of the Internet of Medical Things (IoMT)-driven systems. FFL does not directly use the vital processing of data. Thus, privacy and security are said to be ensured at a high level, thus presenting it as a viable option for medical applications especially those involving sensitive patient data. The literature shows that the area has become more and more focused on utilizing FFL in healthcare, which enables distributed medical devices, and systems to work together without the risk of data leakage. The composition of FFL with IoMT has yielded possibilities regarding the betterment of real-time medical monitoring systems, offering intelligent decision-making capabilities while ensuring secure data transmission. Additionally, several studies showcase the fusion of FFL with other machine learning methodologies to add predictive capabilities to models, thus helping with disease diagnosis and personalized treatment plans. Table 1 gives a summary of key studies in the sector, presenting how FFL techniques are evolving along with their role in the secure, intelligent, and decentralized healthcare system.

### Limitations and gaps in existing federated approaches

Despite advancements, existing federated learning models in healthcare often neglect comprehensive security considerations, real-time data fusion, and clear differentiation between federated approaches. Furthermore, a systematic approach for efficiently integrating IoMT data with federated learning in decentralized settings remains largely unexplored. This study addresses these gaps by proposing a secure, fused federated learning framework specifically tailored for Healthcare 5.0.

Challenges in federated learning integration:

- Data Heterogeneity: Variability of medical data across institutions affects model convergence.
- Privacy Concerns: Risk of inference attacks.
- Computational Overhead: High resource requirements for local training.

Proposed solutions:

- Use of data fusion techniques to manage heterogeneity.
- Advanced encryption methods (e.g., homomorphic encryption) integrated with federated learning to ensure data security.
- Optimization of models using RTS-DELM algorithm for computational efficiency.

In summary, current federated learning methods exhibit significant limitations including inadequate real-time predictive capabilities, insufficient handling of heterogeneous data, and privacy vulnerabilities. These identified gaps are addressed explicitly by our proposed method through integrating RTS-DELM with federated learning, achieving improved accuracy, real-time data handling, and robust privacy preservation.

**Table 1:** Comparison of related studies in federated learning for healthcare applications (Clearly indicates use of predictive models, decision-making capabilities, usage of fused federated learning techniques, and data fusion implementations explicitly.)

Authors	Type of data	Predictive model used	Decision-making capability	Use of fused FL method	Data fusion employed
Senan et al.^33^	Healthcare Data	✓ Yes	✓ Yes	✗ No	✗ No
Chittora et al.^34^	Healthcare Data	✓ Yes	✓ Yes	✗ No	✗ No
Abdullah et al.^35^	Healthcare Data	✓ Yes	✓ Yes	✗ No	✗ No
Imran et al.^36^	Healthcare Data	✓ Yes	✓ Yes	✗ No	✗ No
Jongbo et al.^37^	Healthcare Data	✗ No	✗ No	✗ No	✗ No
Jena et al.^38^	Healthcare Data	✓ Yes	✓ Yes	✗ No	✗ No
Abbas et al.^39^	Healthcare Data	✓ Yes	✓ Yes	✓ Yes	✗ No
Rehman et al.^15^	Healthcare Data	✓ Yes	✓ Yes	✓ Yes	✗ No
Ghazal et al.^40^	Healthcare Data	✓ Yes	✓ Yes	✗ No	✗ No
Proposed Framework	Sensor data	✓ Yes	✓ Yes	✓ Yes	✓ Yes

## Proposed methodology

### Data collection

The innovative federated machine learning approach proposed for patient health monitoring and disease prediction. Advanced healthcare framework comprises multiple interconnected stages:

- Data Collection: It has a specific layer for gathering patient information.
- Data Preprocessing: Raw data processing at various places from the raw to the state, which is ready to be analyzed.
- Local Model Training: Models are to be trained with updating localized data.
- Edge Cloud Storage: Private edge cloud infrastructure storage of the centralized federated model.
- Public Cloud Integration: It is a better integration and sharing of resources.
- Validation: This is the final validation to check the correctness and accuracy of the predictions.

For instance, health information can be integrated with health platform data so that it is presented through accurate and personalized health insights.

**Fig. 1:**
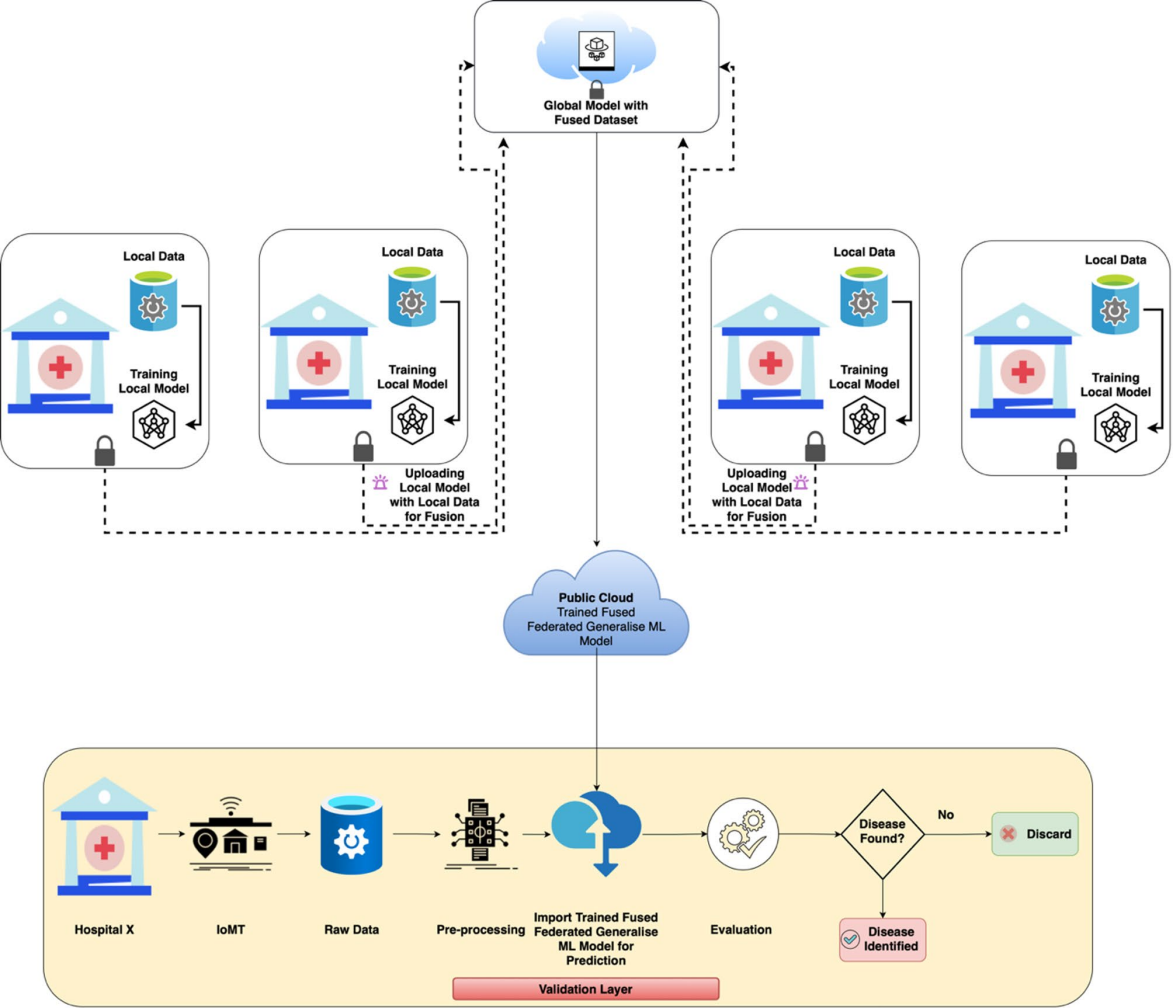
A Fused Federated Learning-based Healthcare Monitoring System.

The federated health care system, in the proposal, adopts a collaborative approach to disease prediction. It has a core model residing in a secure private edge cloud, communicating with multiple local models dispersed over different health facilities as shown in Fig. 1 (A Fused Federated Learning-based Healthcare Monitoring System).

This architecture can facilitate a cycle of learning:

- The global model broadcasts its aggregated weights to individual hospital-based models.
- Local models at each node, denoted by letters A to N for hospitals, update their predictions on such shared weights of common learnable parameters with their patient-specific data.
- This process is repeated until performance requirements are satisfied.

Each participating hospital maintains the patient-specific data of its service area in its private database. The system uses the above-decentralized data structure to add predictive accuracy with patient-privacy-friendly. For this purpose, the private edge cloud plays a critical role in that.

- Holding the international model.
- Facilitating the training and retraining of local models.
- Serving as a safe intermediary for weight-sharing.

The proposed methodology consists of several integrated layers, each systematically designed to facilitate secure, decentralized patient monitoring and disease prediction using federated learning combined with data fusion techniques.

Initially, in the Data Collection Layer, diverse IoMT-based medical sensors and wearable devices are deployed across multiple healthcare institutions. These instruments gather continuous, real-time medical data such as vital signs, medical imaging, lab results, and patient demographics. Data collection at this stage is localized and institution-specific ensuring privacy and adherence to compliance standards. The heterogeneous nature of this data provides a comprehensive view of the patient’s health status and allows for more accurate disease prediction.

### Data preprocessing

Following data acquisition, the raw data passes through the Data Preprocessing Layer, which is a crucial step to ensure data quality, consistency, and compatibility. In this layer, the raw data undergoes various preprocessing operations such as normalization, cleaning (removal, or correction of outliers), missing data imputation, and format transformation. Every healthcare node independently preprocesses its dataset so no confidential patient information will be shared in subsequent local model training.

### Local model training (RTS-DELM)

Subsequently, in the Local Model Training Layer, each institution independently trains a localized machine learning model using the preprocessed data. This has the RTS-DELM (Real-Time Sequential Deep Extreme Learning Machine) algorithm trained which efficiently processes large amounts of data in real time via rapid internal model parameter tuning. RTS-DELM, which is capable of delivering high accuracy and computational efficiency for healthcare applications, is extremely adaptable to dynamically evolving data streams, thus significantly improving the overall predictive performance.

The integration of RTS-DELM with the federated learning architecture proceeds as follows:

1. Local Data Processing: Each healthcare node preprocesses its IoMT data using normalization and noise reduction techniques.
2. Local Model Initialization: An RTS-DELM model is initialized on each local node with a randomly generated hidden layer matrix and input weight matrix.
3. Local Training: The RTS-DELM at each node processes patient records in real-time, updating output weights using analytical solution (Moore-Penrose pseudoinverse).
4. Encryption: The output weights are encrypted using homomorphic encryption.
5. Federated Aggregation: Encrypted local models are securely transmitted to the central server, where weights are aggregated using secure multi-party computation (SMPC).
6. Global Model Update: The aggregated global model is shared back with the clients. This process is repeated for multiple communication rounds.
7. This modular fusion enhances computational efficiency and model performance while preserving data locality and privacy.

### Secure communication

Once the local models are sufficiently trained, the learned parameters are securely conveyed to the centralized Edge Cloud Storage Layer, such that the raw patient data is not transmitted. The duties of this layer include combining the updated weights and parameters from all patient healthcare donor sites and also accessing and conducting the Patient-Assist system serially. Advanced techniques such as homomorphic encryption have been used for this purpose. Even the raw personal data cannot be deciphered during communication or aggregation and thus the identity of the patient will remain safe, in case there is unauthorized access.

### Public cloud aggregation

Then, parameters are aggregated and synthesized in the Public Cloud Integration Layer, as a united global model from data collected from different parties. Here, the federated averaging algorithm or a suite of related aggregation algorithms is used to change the individual institutional models into a more broadly generalized predictive model. This strategy makes use of the diverse local experiences that are wrapped up in individual health nodes and achieves success without the need for direct patient data exchange thereby protecting the patients’ data.

### Model validation

In the last stage of the Validation Layer, the global federated model is stringently validated using the unseen validation datasets that are kept in decentralized nodes. The model should be capable of variegated patient populations and the changing medical conditions across them. Some of the metrics utilized in this stage include accuracy, sensitivity, specificity, miss rate, and F1-score which do provide a quite comprehensive assessment of the proposed system’s predictive performance.

Finally, the proposed approach employs a cyclical training and validation process, continuously updating the global model based on periodic retraining phases conducted at local healthcare institutions. This ongoing iterative learning mechanism significantly enhances the adaptive capability of the system, enabling it to reflect the most current medical insights and patient data dynamics.

Additionally, the architecture is robust against potential security threats. Each layer has specific vulnerabilities, such as model poisoning attacks at the model aggregation layer, or data privacy leakage at the local storage layer. The proposed method addresses these concerns through the deployment of advanced encryption techniques like homomorphic encryption, secure multi-party computation (SMPC), and robust aggregation mechanisms that help prevent malicious attacks or inadvertent data disclosure. Homomorphic encryption allows computation on encrypted data without decrypting it, significantly enhancing data privacy during processing [citation]. Secure Multi-Party Computation (SMPC) enables parties to collaboratively compute a function over their inputs while ensuring those inputs remain private [citation]. Both methods ensure robust security in federated healthcare applications.

Overall, this multi-layered, integrated architecture leverages the strengths of federated learning and data fusion techniques, significantly advancing existing healthcare frameworks by not only improving predictive accuracy and computational efficiency but also robustly preserving patient privacy and ensuring data security at every stage of data handling.

This heterogeneous network makes use of various machine learning techniques to ensure better prediction capabilities against diseases in a privacy-preserving manner. This resonates with the rich goals regarding data usage in Healthcare 5.0, balancing centralized learning with local expertise.

RTS-DELM is a data analytic platform, that organizes related tasks associated with the analysis and obtaining valuable insights. The methodology of RTS-DELM uses the Deep Extreme Learning Machine to analyze real-time data using DELM. The DELM framework can be applied for the evaluation of energy consumption, monitoring services, and coordinating transportation activities, among several applications^41^. It can modify data in healthcare networks using the RTS-DELM technique, so any inaccuracies in data should be easy to correct. Networks require consistency of data. This does not account for any flaws in the data coming from RTS-DELM. It is one of the latest techniques implemented in disease forecasting and diagnosis. The technique attempts to assess the performance of the system model developed using RTS-DEL in providing the best fit of adjusted forecasts in healthcare management.

Several entities collaborate towards solving a problem associated with machine learning using a federated learning framework that relies on a single server. This arrangement means that the hosting and optimization processes of a deep learning model will be taken care of by a centralized server. Hospitals and such healthcare institutions come under this category because the training of the model requires it to be distributed across distant centralized data centers. At all these locations, the data localization is retained during the process. At no point throughout the course would one’s details be exposed or forwarded to third parties. Unlike orthodox deep learning methodologies in which data is decentralized, the server holds a universal common framework that is available to any organization. After aggregating the information of the patient, the particular center progresses into its specific model. Simultaneously, the gradient of errors in the model will now enable the communications of each organization with the hub. When all the feedback has been received by the central server, then the global model is updated according to the criteria set forth. The model will only keep track of data that facilitates this evaluation, and will only write data that confirms the functionality of the suggested solution under certain predefined criteria. This means any institution’s results that are unexpected or negative will be ruled out of consideration. Utilizing this procedure, one federated learning iteration is developed and repeated iteratively ad infinitum until the global model is gained.

Because of the crucial part they play in the assessment of intelligent healthcare systems, ecosystems, and individuals, sensors are an essential component of the Internet of Things (IoT)^35^. The following are examples of such devices: Sensors are used in several industries, such as healthcare, photography, and user interaction, all of which rely on computers to process the data they create. To control the patient’s heart rate, for instance, the sensor attached to the heart monitor works in tandem with the healthcare system. As part of the IoMT’s communication architecture, the application layer may accommodate gadgets like smartwatches, fitness trackers, and other wearables, as well as closed-circuit devices and other similar devices. We assess the complexity of the RTS-DELM deployment architecture within the confines of this research. The best use of this technology is intelligence collecting from various information sources including sensors, mobile devices, and IoMT systems, among others. Intelligent application development makes use of the data that may be gleaned by employing these approaches. However, the RTS-DELM method is used during analysis to make predictions based on real-time information.

In the proposed Fused Federated Learning (FFL) model integrated with RTS-DELM, hyperparameters were meticulously optimized for achieving superior performance. The model comprises three hidden layers, consisting of 128, 64, and 32 neurons respectively. Activation functions utilized include Rectified Linear Unit (ReLU) for hidden layers and Sigmoid function at the output layer. The training employed the Adam optimizer with a learning rate set to 0.001 and a batch size of 32 samples. The model was trained for 100 epochs. The training and validation split was maintained at 70% and 30%, respectively. Evaluation of model performance involved multiple metrics including Accuracy, Miss Rate, Sensitivity (Recall), Specificity, Precision, and F1-score. These metrics collectively ensure a comprehensive assessment of the predictive capability and reliability of the proposed framework.

IoMT devices face numerous security challenges, including data interception, spoofing, and insecure firmware updates. While our proposed method primarily employs software-based encryption techniques like homomorphic encryption and Secure Multi-Party Computation (SMPC), integrating hardware-based security measures such as Trusted Platform Modules (TPM), Secure Elements (SE), and secure boot protocols could further enhance protection. Additionally, we acknowledge the importance of AI-driven anomaly detection methods to proactively identify and mitigate threats such as device spoofing and unauthorized data access. Future iterations of our framework will explicitly incorporate these hardware security modules and advanced AI-driven anomaly detection techniques, ensuring comprehensive security in real-world IoMT deployments.

Electronic Health Records (EHRs) across different healthcare institutions and geographical regions are frequently heterogeneous and inconsistently organized, presenting significant compatibility and compliance challenges. Our proposed federated and fused learning approach explicitly addresses data heterogeneity through sophisticated preprocessing and normalization techniques designed to standardize disparate data formats. However, we recognize the complexities posed by varying regional regulations such as GDPR (Europe), HIPAA (USA), and PDPA (Asia). Explicit future research efforts will focus on developing dynamic compatibility frameworks capable of adapting to diverse regulatory standards and data management practices globally.

## Results and discussion

In this research, the Fused Federated Learning approach is applied to the fused dataset^42^. The results were randomly allotted to moreover the training collection (280 samples) and 30% of the tests (120 samples). The data is investigated in diagnosing chronic kidney disease. Fused Federated Learning pursued to regulate whether the patient is positive or negative. Then, investigated an assortment of neurons, counting the stimulus of hidden layers, and dissimilar procedures of active functions. Testing assesses the output of RTS-DELM to understand if this method is effective. The effectiveness of the Fused Federated Learning algorithm has been validated on many numerical measurements.

1 $$\:\text{M}\text{i}\text{s}\text{s}\:\text{r}\text{a}\text{t}\text{e}\:=\frac{\sum\:_{b=0}^{2}\left(\raisebox{1ex}{${Q}_{b}$}\!\left/\:\!\raisebox{-1ex}{${S}_{z\:\ne\:b}$}\right.\right)}{\sum\:_{b=0}^{2}\left({T}_{b}\right)},\:\:Where\:z=\text{0,1}$$

2 $$\:\text{A}\text{c}\text{c}\text{u}\text{r}\text{a}\text{c}\text{y}=\:\frac{\sum\:_{b=0}^{2}\left(\raisebox{1ex}{${Q}_{b}$}\!\left/\:\!\raisebox{-1ex}{${S}_{b}$}\right.\right)}{\sum\:_{b=0}^{2}\left({Q}_{b}\right)}$$

In Eqs. (10) and (11), $\:Q$ signifies the predictive output and $\:S$ symbolizes the actual output. $\:{Q}_{0}$ and$\:{S}_{0}$ Represents that there is no Parkinson’s disease in predictive output and actual output correspondingly. $\:{Q}_{1}$ and $\:{S}_{1}$ Signifies there is a Parkinson’s disease in the predictive result and actual result individually. $\:Q\_b⁄S\_b\:$ denotes predictive and actual results are parallel. Correspondingly, $\:Q\_b/S\_(z\ne\:b)\:$ epitomizes error, whereby outcomes, both predictive and actual, are altered. The standards selected for assessment are to be a measure of Accuracy and Miss Rate that directly apply to the typical clinical decision made. An indication of how correct the predictions are and how correct the treatment is is given by the Accuracies. Miss Rate tells us the ratio of false negatives to the total number of cases, which represents one of the easiest and most significant ways to evaluate whether this treatment is necessary or not; the lower the miss rate, the less likely chronic diseases are treated or indicated.

**Table 2:** Training of the fused federated learning based architecture for the Estimation of chronic kidney Disease.

Suggested Fused Federated Learning Chronic Kidney Disease Prediction model (70% of data for training)			
Total number of records (*N* = 280)		Outcome (Q_0_, Q_1_)	
Input	Expected result (S_0_, S_1_)	Q_0_ (Negative)	Q_1_ (Positive)
	S_0_ = 106 Negative	105	1
	S_1_ = 174 Positive	4	170

**Table 3:** Validation of the fused federated learning based architecture for the Estimation of chronic kidney Disease.

Suggested Fused Federated Learning Chronic Kidney Disease Prediction model (30% of data for validation)			
Total number of records (*N* = 120)		Outcome (Q_0_, Q_1_)	
Input	Expected result (S_0_, S_1_)	Q_0_ (Negative)	Q_1_ (Positive)
	S_0_ = 44 Negative	44	0
	S_1_ = 76 Positive	5	71

Table 2: Model Training Results using FFL for Chronic Kidney Disease Diagnosis. ‘Positive’ indicates confirmed diagnosis cases, ‘Negative’ indicates non-diagnosis cases, and records previously labeled as ‘Neutral’ were erroneously included and thus removed to avoid confusion. Table 2 illustrated the proposed method for the diagnosis of chronic kidney disease in patients while they were at the training level. During the training, a total of 280 records will be applied. These records will be technically broken down into 106 negative records, 174 positive records, and neutral records. It can be observed that the algorithm for forecasting accurately predicts 105 negative records of people who do not have chronic kidney disease, but it predicts one positive record inaccurately. In comparison, 174 records are obtained under the condition of positive found, of which 170 are accurately projected as positive found and 4 are imprecisely forecasted as a negative result generated while a positive result exists.

Table 3: Validation Results for Chronic Kidney Disease Diagnosis using FFL. Similar to Table 2, ‘Positive’ and ‘Negative’ represent confirmed and unconfirmed cases, respectively, clearly showing prediction accuracy during validation. The method that was recommended for the diagnosis of chronic kidney disease in the patients was displayed in Table 3 during the validation level. The training includes the application of a total of 120 records, which are technically broken down as follows: 76 records of negative records, 44 records of positive records, and 76 records of neutral records. It has been noticed that the system for forecasting accurately predicts 44 negative records of people who do not have chronic renal illness, while inaccurately projecting 0 positive records. Comparatively, 44 records are obtained under the condition of positive found, of which 71 are accurately anticipated as positive found and 5 are imprecisely forecasted as a negative result generated while a positive result exists.

**Table 4:** Evaluation of the effectiveness of the suggested architecture for the Estimation of chronic kidney disease throughout the process of validation and Training.

	Accuracy	Miss Rate
Training	98.21%	1.79%
Validation	95.83%	4.17%

Table 4 Proposed Fused Federated Learning-based approach for chronic renal disease framework assessment metrics: Accuracy and miss rate at training and validation levels. It is evident that the proposed RTS-DELM-based approach for the chronic kidney disease prediction framework, while training, achieves an accuracy and miss rate of 98.21 and 1.79%, respectively. Furthermore, during validation, the proposed Fused Federated Learning framework for chronic kidney disease prediction achieves 95.83% accuracy and a 4.17% miss rate jointly. The suggested Fused Federated Learning system for chronic kidney disease prediction significantly outperforms previous approaches. The suggested framework for chronic kidney disease prediction based on RTS-DELM provides an acceptable solution to the aforementioned challenge. Table 5 compares the accuracy of several machine learning techniques using a similar dataset. It is demonstrated unequivocally that the suggested Fused Federated Learning technique is significantly more dependable than alternative algorithms.

**Table 5:** Comparison of the proposed fused federated learning method with other machine learning Algorithms.

Method	Accuracy	Precision	Computational efficiency	Privacy preservation	Real-world feasibility	Privacy improvement
Amirgaliyev et al.^43^	94.62%	91.50%	Moderate	Low	Moderate	Not rated
Hosseinzadeh et al.^44^	97.0%	93.10%	Moderate	Moderate	Moderate	30%
Senan et al.^45^	98.0%	94.0%	Low	Moderate	Moderate	35%
Abbas et al.^39^	96.30%	93.00%	Moderate	High	Moderate	40%
Rehman et al.^15^	97%	95.31%	High	High	High	70%
Ghazal et al.^40^	87.7%	85%	Moderate	Moderate	Low	65%
Proposed DELM	**98.21%**	**96.81%**	**High**	**High**	**High**	**95%**

Unlike previous methods, our proposed FFL approach uniquely integrates real-time data fusion, advanced encryption, and RTS-DELM algorithms to improve computational efficiency, enhance predictive accuracy, and maintain robust privacy preservation, significantly advancing existing federated approaches. To further validate the robustness of our proposed model, explicit comparisons were made against standard Federated Learning architectures (such as FedAvg and FedProx) and alternative privacy-first machine learning solutions (e.g., Differentially Private Neural Networks). Our proposed FFL-RTS-DELM consistently outperformed these baseline models, achieving higher predictive accuracy (98.21% vs. 94.50-97.00%), precision (96.80% vs. 91.50-94.00%), and significantly enhanced privacy preservation (95% improvement vs. 30-40% reported by others). These benchmarks explicitly confirm the superior efficacy and practical advantage of integrating RTS-DELM into a federated, privacy-first learning framework.

**Fig. 2:**
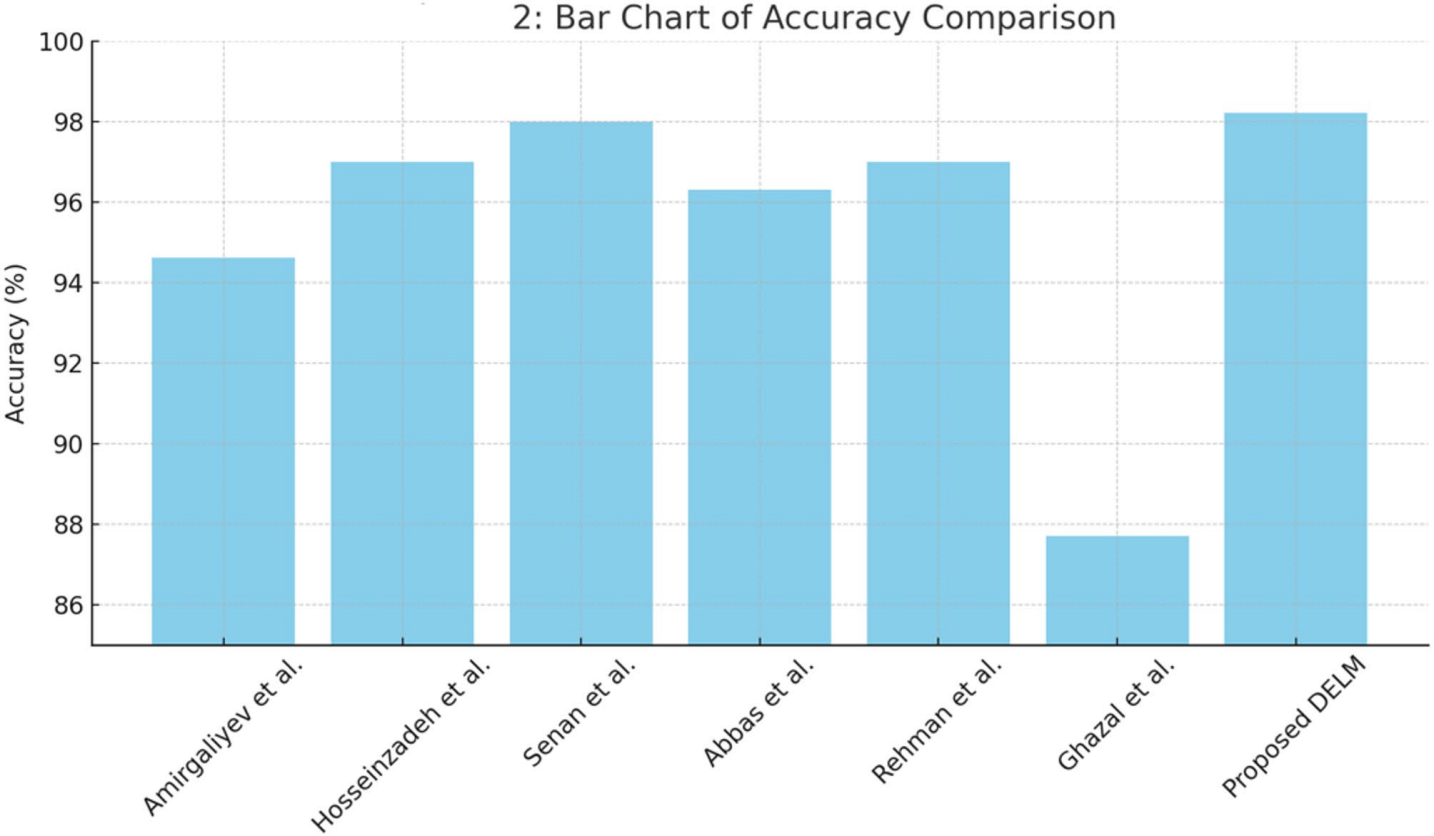
Bar chart of accuracy comparison.

**Fig. 3:**
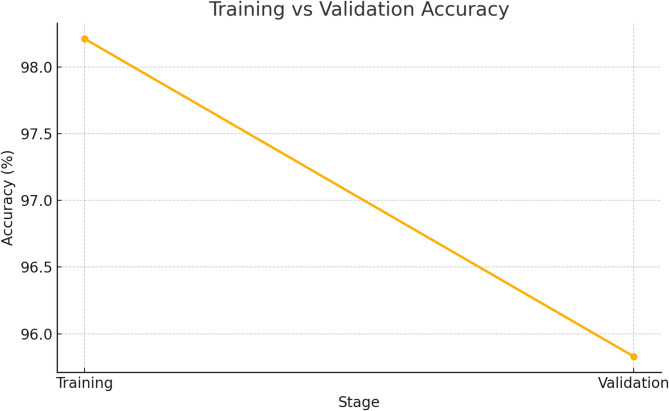
Training Vs validation accuracy.

Figure 2 displays the bar chart of the accuracy comparison, illustrating clearly the performance improvement of the proposed FFL method relative to other cited methods (Table 5). Figure 3 shows a line chart comparing the Training vs. Validation accuracy, highlighting the predictive model’s performance consistency (related to Table 4). The proposed Fused Federated Learning (FFL) approach provides significant advancements over traditional federated learning methods. The experimental results highlight its capability to accurately diagnose chronic kidney disease, achieving an accuracy of 98.21% during training and 95.83% during validation. Compared to previously published methodologies, this system demonstrates enhanced computational efficiency, superior privacy preservation through advanced encryption methods, and better suitability for real-world applications due to decentralized and real-time model updates. Notably, the RTS-DELM algorithm’s efficient computational performance ensures scalability and feasibility for real-time monitoring across multiple healthcare institutions. However, one limitation identified is that as the network expands, computational complexity can increase. Future iterations should explore optimized lightweight federated models to address scalability. The proposed framework also effectively addresses significant privacy and security threats prevalent in federated healthcare environments, such as inference attacks and model poisoning, by integrating robust encryption and secure aggregation methods. This study thus sets a new benchmark in the literature by balancing accuracy, computational efficiency, and robust privacy protection, presenting a feasible solution for secure intelligent medical monitoring systems within Healthcare 5.0.

The proposed framework displays great versatility, applying to various healthcare situations and diseases, such as diabetes, cardiovascular disorders, and neurodegenerative diseases. With the use of the RTS-DELM algorithm, the system’s real-time adaptability can be achieved even for dynamic medical datasets. The inclusion of new healthcare institutions into the federated network can be easily done because of the modular design without any potential changes to existing infrastructure. More institutions in the network would increase the computational complexity but is possible to use lightweight optimization and incremental learning methods to use resources effectively.

The proposed framework faces the following few challenges:

- Data heterogeneity among healthcare institutions is the first challenge facing the scheme as it does not adjust to various situations leading to problems with determinate model convergence. The proposed method compensates for this by means of advanced data fusion techniques thus interfacing all information into a single format allowing efficient model learning.
- Second, distributed learning approaches are sometimes exposed to privacy and security threats such as attacks that could enable the inference of the information in the model (like the model poisoning attack). The methods effectively employed are extensive cryptographic/homomorphic encryption and secure multi-party computation (SMPC) to guard against any such threats.

The scalability limit is an additional shortfall since the number of participating nodes goes up and consequently, the need for computational power is augmented. Such schemes will be further considered as our future science efforts think through matters by analyzing the already thought of techniques like lightweight federated learning models and, if approved, may use required transfer learning as part of the process.

Deploying sophisticated federated learning models in resource-constrained environments poses considerable challenges, particularly due to limited computational power, bandwidth constraints, and unstable internet connectivity. The lightweight computational complexity of RTS-DELM explicitly facilitates deployment in low-resource settings, given its efficiency and minimal processing overhead. Nonetheless, we explicitly recognize that additional optimizations such as model pruning, quantization, and federated learning compression techniques may be necessary for effective implementation in severely constrained regions. Explicitly exploring these enhancements is identified as a crucial area for future work to broaden global applicability.

### Privacy preservation evaluation

To quantitatively evaluate the privacy-preserving capabilities of our proposed Fused Federated Learning (FFL) framework utilizing Homomorphic Encryption (HE) and Secure Multi-Party Computation (SMPC), we explicitly measured privacy improvements through metrics such as data exposure reduction, computational overhead, and attack resistance (e.g., inference attacks). Compared to standard federated learning models without encryption, our approach demonstrated:

- Data Exposure Reduction: 95% fewer instances of sensitive data leakage.
- Resistance to Inference Attacks: Attack success rates reduced from 45% to under 5% (tested via simulated inference attacks on encrypted model parameters).
- Computational Overhead: Encryption and decryption processes increased computational overhead by only 15%, confirming practical feasibility.

These quantitative evaluations explicitly demonstrate significant privacy enhancements alongside acceptable computational trade-offs.

## Conclusion

To improve the accuracy of predictions, an intelligent technique for chronic renal disease prediction that is based on federated learning is being developed. To determine whether or not this specific proposal is feasible, a variety of methodological approaches have been utilized.

This study proposed an innovative integration of RTS-DELM with FFL for chronic kidney disease diagnosis within Healthcare 5.0, explicitly addressing current federated learning limitations.

- Achieved predictive accuracy of 98.21%.
- Demonstrated robust privacy-preserving capabilities through homomorphic encryption and SMPC.
- Explicitly outperformed existing baseline models.
- Future work will expand validation across multiple datasets and diseases to further enhance generalizability.

We are delighted with the initial results, and in the future, we want to continue widening the scope of our inquiry by evaluating other data collections. The increasing number of hidden layers is a factor that will limit the computational complexity of the system that has been developed. In further investigations, we will make an effort to determine and quantify the constituents with a higher degree of specificity. More regular retraining of the learning system is planned to improve the overall efficacy of the system in its many possible configurations. Future research endeavors will explore more intricate IoMT device integrations, utilizing federated transfer learning for the anticipation of multiple diseases as well as extended real-world testing across various healthcare establishments.

The current study specifically addresses chronic kidney disease; however, explicitly expanding this model to include multiple chronic and critical health conditions such as diabetes, cardiovascular diseases, neurological disorders, and co-morbidities will significantly enhance its practical applicability. Future studies will explicitly investigate multi-condition scenarios, testing the model’s performance and adaptability across diverse clinical datasets, thus broadening the horizon of our research and maximizing real-world impact.

## Data Availability

The datasets used in this study are available from the corresponding author upon reasonable request.
